# Osteochondral Regeneration Induced by TGF-β Loaded Photo Cross-Linked Hyaluronic Acid Hydrogel Infiltrated in Fused Deposition-Manufactured Composite Scaffold of Hydroxyapatite and Poly (Ethylene Glycol)-Block-Poly(ε-Caprolactone)

**DOI:** 10.3390/polym9050182

**Published:** 2017-05-20

**Authors:** Yi-Ho Hsieh, Ming-Fa Hsieh, Chih-Hsiang Fang, Cho-Pei Jiang, Bojain Lin, Hung-Maan Lee

**Affiliations:** 1 Department of Biomedical Engineering, Chung Yuan Christian University, 200 Chung Pei Road, Chung-Li District, Taoyuan City 320, Taiwan; dilantin11@gmail.com (Y.-H.H.); mfhsieh@cycu.edu.tw (M.-F.H.); danny07291991@hotmail.com (C.-H.F.); linbojain@gmail.com (B.L.); 2 Department of Orthopedics, Min-Sheng General Hospital, 168, ChingKuo Rd, Taoyuan 330, Taiwan; 3 Department of Power Mechanical Engineering, National Formosa University, Yunlin County 632, Taiwan; kasu23@gmail.com; 4 Department of Orthopedics, Taoyuan Armed Forces General Hospital, No. 168, Zhongxing Road, Longtan District, Taoyuan City 325, Taiwan; 5 Department of Orthopedics, Hualien Tzu Chi General Hospital, No. 707, Sec. 3, Chung Yang Rd, Hualien 970, Taiwan

**Keywords:** poly(ε-caprolactone), osteoarthritis, cartilage, scaffold, hydrogel, fused deposition manufacturing

## Abstract

The aim of this study was to report the fabrication of porous scaffolds with pre-designed internal pores using a fused deposition modeling (FDM) method. Polycaprolactone (PCL) is a suitable material for the FDM method due to the fact it can be melted and has adequate flexural modulus and strength to be formed into a filament. In our study, the filaments of methoxy poly(ethylene glycol)-block-poly(ε-caprolactone) having terminal groups of carboxylic acid were deposited layer by layer. Raw materials having a weight ratio of hydroxyapatite (HAp) to polymer of 1:2 was used for FDM. To promote cell adhesion, amino groups of the Arg-Gly-Asp(RGD) peptide were condensed with the carboxylic groups on the surface of the fabricated scaffold. Then the scaffold was infiltrated with hydrogel of glycidyl methacrylate hyaluronic acid loading with 10 ng/mL of TGF-β1 and photo cross-linked on the top of the scaffolds. Serious tests of mechanical and biological properties were performed in vitro. HAp was found to significantly increase the compressive strength of the porous scaffolds. Among three orientations of the filaments, the lay down pattern 0°/90° scaffolds exhibited the highest compressive strength. Fluorescent staining of the cytoskeleton found that the osteoblast-like cells and stem cells well spread on RGD-modified PEG-PCL film indicating a favorable surface for the proliferation of cells. An in vivo test was performed on rabbit knee. The histological sections indicated that the bone and cartilage defects produced in the knees were fully healed 12 weeks after the implantation of the TGF-β1 loaded hydrogel and scaffolds, and regenerated cartilage was hyaline cartilage as indicated by alcian blue and periodic acid-schiff double staining.

## 1. Introduction

Articular cartilage is hyaline cartilage covering the articular surface of bones. Due to its avascular characteristic, once injured, articular cartilage has a poor self-repair ability. Treatment includes conservative treatment and surgical approaches, but there is currently no effective treatment for this disease. Bone marrow stimulation (microfracture surgery) could produce cartilage formation, but the newly-formed cartilage is fibrocartilage instead of hyaline cartilage. Several methods of chondrocyte implantation were used to treat cartilage defects in humans, though implanted grafts do not provide mechanical stability and various side effects lead to procedure failure [1,2]. Osteochondral plug transplantation (mosaicplasty) has been developed but the limitations of this technique include donor site morbidity and limited availability of grafts that can be harvested. The long-term outcome is constrained by the age and gender of the patient and the size of the wound [3,4,5].

Various tissue-engineering approaches were developed using natural or synthetic biomaterials as scaffolds for cell growth. The porous scaffolds are traditionally fabricated by the particulate leaching method, adding pore-forming agents and their combinations [6]. However, the pore size and interconnection of pores could not be precisely controlled throughout the entire scaffold. Recently, rapid prototyping (RP) technology has emerged to produce custom-made scaffolds and provide a new method to control the internal architecture of the scaffolds for cell growth [7,8]. Among the natural biopolymers and synthetic polymers, poly(ε-caprolactone) (PCL) has been widely studied for 3D printing applications because of the longer biodegradation time in the human body as compared to poly(lactide-co-glycolide) [9,10,11,12]. Woodfield et al. employed FDM to fabricate articulating scaffolds having a natural curvature of the surface for fitting into the individual patient’s knee [13]. They showed that chondrocytes grew into scaffolds and secreted the ECM of cartilage [14]. Boere et al. showed that chondrocytes could be embedded into 3D-fabricated polymeric scaffolds, showing significant deposition of ECM in their constructs, both in vitro and in vivo [15]. Shao et al. implanted PCL scaffolds into cancellous bone while fibrin glue was applied on top of the cartilage surface. Therefore, PCL scaffolds are a promising matrix for bone regeneration and fibrin glue for cartilage regeneration [16].

The successful interaction between the cell and the surface of the biomaterial is important to the scaffolds. The PCL scaffold has good biocompatibility and biodegradability, but lacks biological factors on its surface, leading cells to not easily attach and grow. RGD (Arg-Gly-Asp) is a peptide for biopolymer functionalization and can trigger cell lines to adhere and proliferate on the polymer’s surface. Characterizing the chemical modification and, therefore, the extent of RGD (Arg-Gly-Asp) at the polymer’s surface can significantly promote cell adhesion and proliferation [17,18].

Hydrogels can simulate the microenvironments of natural tissues, encapsulate cells homogeneously, and deliver drugs, which makes them attractive for cartilage tissue engineering [19,20]. Hydrogels loaded with growth factor could ideally fit into irregular cartilage wounds while releasing the growth factor [21], and three-dimensional networks of hydrogels are known to proliferate and differentiate mesenchymal stem cells [22]. For example, Guo et al. Used a bi-layered hydrogel of oligo(poly(ethylene glycol) fumarate to encapsulate TGF-β loaded gelatin microparticles for chondrogenic differentiation and osteochondral regeneration [23,24]. However, the osteochondral defects were not healed using their biphasic scaffolds in vivo. For delivery of the growth factors, hyaluronic acid (HA), among other hydrogels, has been grafted with glycidyl methacrylate to bestow a HA photo cross-linkable property [25,26]. The light-cured HA hydrogel displays various properties, such as sealant of irregular defects of cartilage [27], the carrier of growth factors [28], ECM to enhance MSC differentiation [29], or to maintain the differentiated phenotype [30].

Recently, tissue engineering for articular cartilage repair has focused on biphasic scaffolds [31]. Cartilage damage involves not only the articular surface of the knee, but also the subchondral bone. Currently, the performance of scaffolds combining with hydrogels reported so far with respect to damaged cartilage is not satisfactory. Therefore, we aimed to fabricate a biphasic scaffold with two layers for osteochondral regeneration. The bottom layer is a fused-deposited methoxy poly(ethylene glycol)-block-poly(β-caprolactone) scaffold (mPEG-PCL), which was first modified by adding hydroxyapatite (HAp) powder and grafting RGD peptide to the surface of the scaffold. The surface layer of the mPEG-PCL scaffold was infiltrated with glycidyl methacrylate-hyaluronic acid (GMHA) hydrogel containing transforming growth factor TGF-β1 for cartilage healing.

## 2. Materials and Methods

### 2.1. Materials

Poly(ethylene glycol) (PEG, number-average molecular weight (*M*_n_ = 4000 g·mol^−1^) was obtained from Showa (Tokyo, Japan); ε-caprolactone (ε-CL), stannous octoate (Sn(Oct)_2)_, 1,6-Diphenyl-1,3,5-hexatriene (DPH), dimethyl sulfoxide (DMSO), 3-(4,5-Dimethylthiazol-2-yl)-2,5-diphenyltetrazolium bromide (MTT), phosphotungstic acid (PTA), 2-Hydroxy-2-methylpropiophenone, Arg-Gly-Asp (RGD peptide), Hydroxyapatite, Noble agar, potassium phosphate monobasic, potassium bromide (KBr), sodium chloride, and sodium phosphate were all obtained from Sigma-Aldrich Chem. Inc. (St. Louis, MO, USA). 1,4-dioxane, Acetone, acetonitrile (ACN), Triethylamine, D-chloroform (CDCl3), dichloromethane, ethyl ether, ethanol, hexane, and tetrahydrofuran were provided by ECHO Chemicals (Miaoli, Taiwan).

### 2.2. Synthesis of Biopolymers

**Figure 1:**
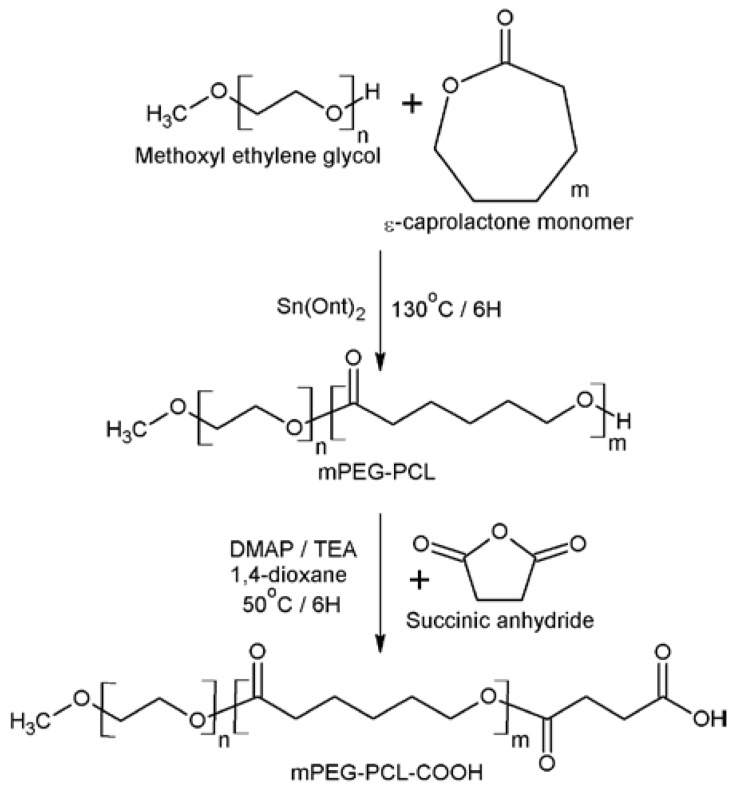
Synthesis scheme of the mPEG-PCL-COOH diblock copolymer.

In this study, biopolymer synthesis is divided into two parts. The first part synthesized the diblock copolymers (mPEG-PCL), and the second part modified the diblock copolymers to terminal function groups (mPEG-PCL-COOH) (Figure 1), as in our previous reports [9]. In brief, the mPEG-PCL diblock copolymers were synthesized by ring-opening polymerization of ε-CL using mPEG as the macro-initiator and Sn(Oct)_2_ serving as a catalyst. The hydroxyl group of mPEG-PCL was modified to a carboxylic acid group and was connected with mPEG-PCL diblock copolymers. The theoretical molecular weight of mPEG-PCL is defined as 9450 Da whereas the molecular weight of mPEG is 550 Da.

### 2.3. Characterization of Biopolymers

Thermal properties were measured by differential scanning calorimetry (Jade DSC, PerkinElmer, Waltham, MA, USA). Samples with weights ranging from 5 to 10 mg were put into the aluminum pans with two series of heating and cooling. The samples were heated up to 150 °C and cooled down to 20 °C. All operations were conducted at the rate of 10 °C/min. The vibrational spectra of Fourier transform infrared spectroscopy were carried out on a FTIR 410 (JASCO, Tokyo, Japan) ranging from 4000 to 400 cm^−1^. The powdery polymers were mixed with KBr powder and compressed into a disk for FTIR measurements. Two-hundred fifty-six (256) scans were performed in all specimens, and the spectrum was recorded. The average molecular weight (*M*_w_) and the polydispersity (PDI, *M*_w_*/M*_n_) of the polymers were determined by Gel Permeation Chromatography (GPC 270, Viscotek, Malvern, UK) connected with a refractive index detector. Tetrahydrofuran (THF) was used as an eluent. The molecular weight was calculated using standard polystyrene samples as references. The molecular structure of the polymer was determined by nuclear magnetic resonance spectrophotometry (500 MHz, Bruker, MA, USA). The ^1^H NMR spectra of the block biopolymers were recorded, using D-chloroform (CDCl_3_) as the solvent. Further, the average molecular weight and grafting percentage of the biopolymers were calculated based on the spectra.

### 2.4. Fabrication of Porous Scaffolds of mPEG-PCL-COOH/HAp

The powders of polymer and HAp were mixed at a weight ratio of 2:1 in a glass container. To ensure thorough mixing of the powders, the mixing was performed using a homemade roller. The fused deposition modeling of the mixed powder was performed in a homemade air pressure-aided deposition system. It consists of a sample compartment (stainless steel cylinder) surrounded by a heating system (heater, controller, and nozzle), the air pressure pump, three-axes motors, and self-developed software (NI LabView).

**Figure 2:**
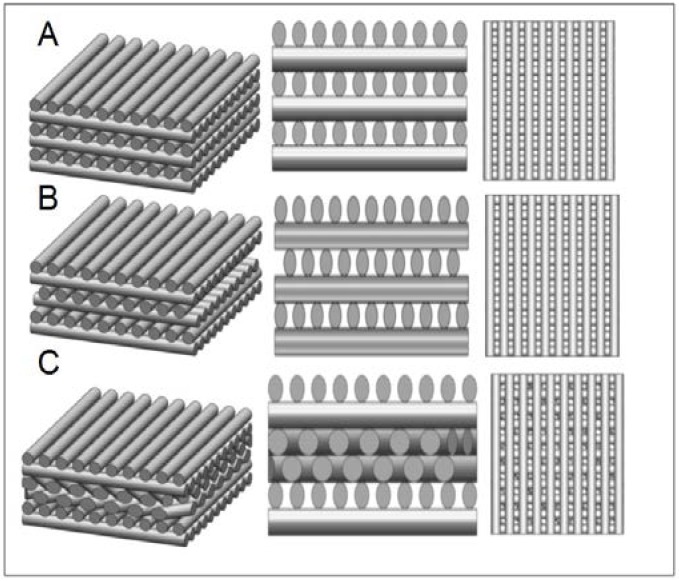
The structure of (**A**) lay-down patterns 0°/90°(90ECR/90ECR); (**B**) staggered 0°/90°(SECR/SECR); and (**C**) 0°/90°/+45°/−45° (45ECR/45ECR) scaffold designs.

The mixed powder was heated up to 60 °C for complete melting. Afterward, a pressure of 15 psi was applied to extrude the molten sample from an 18-gauge nozzle onto a computer-controlled *x*-*y*-*z* table. The extruded filament was laid down layer-by-layer in three different layer orientations: 0°/90°, staggered 0°/90°, and 0°/90°/+45°/−45° at a speed of 4 mm/s. The structure of scaffolds was computer-designed (Figure 2). The average pore size measurements of the scaffolds can be found in our previous study [9]. In our study, scaffold surfaces were modified by RGD peptide grafting. RGD peptide is known to enhance cell adhesion by binding the intergen αvβ3 in the cell membrane [32]. In brief, polymers created COOH functional groups, and were prepared by our home-made RP machine. Scaffolds were immersed in a solution of dimethylaminopropyl-3-ethylcarbodiimide hydrochloride (EDC, 0.2 M) + *N*-hydroxysuccinimide (NHS, 0.1 M) in (2-(*N*-morpholino)-ethanesulfonic acid (MES buffer, 0.1 M in d^2^H_2_O) for 30 min to activate the hydroxyl groups and then rinsed in d^2^H_2_O. After that, immobilization of RGD peptides was achieved with a solution of RGD peptides (10^−3^ M) by phosphate buffered solution (PBS) for 24 h at 4 °C. After grafting, the scaffolds were rinsed with d^2^H_2_O (100 mL) for 10 min in order to remove non-grafted peptides.

### 2.5. Determination of Inorganic Components in the Scaffolds

Thermogravimetric analysis was used to determine the mass change of a scaffold as a function of temperature or time. This analysis confirmed that the composite contained the correct amounts of inorganic components, e.g., hydroxyapatite. Samples with weights ranging from 10 to 20 mg were put into the furnace with one series of heating. The sample was heated from 25 °C to 900 °C. All operations were conducted at the rate of 10 °C/min.

### 2.6. Mechanical Testing of the Scaffolds

Compression tests were conducted on all structures of the fabricated scaffolds. Compressive properties of the scaffolds were measured by using the material compression test system (Chun Yen Testing Machines CY-6040A4). Specimen dimensions were 10 mm in length, 10 mm in width, and 4 mm in height. Conducted with a cross-head displacement speed of 1 mm/min, compressive strengths were obtained from the testing system and Young’s modulus were calculated.

### 2.7. In Vitro Degradation

Rapid-prototyped scaffolds (10 × 10 × 4 mm) were prepared into rectangle shape for degradation testing. In vitro degradation study was carried out in PBS solution according to the standard protocol ASTM F1635 [33]. The RP scaffolds grafting RGD peptide were sterilized under UV light irradiation for 2 h, placed in 100 mL glass container containing 50 mL of PBS and incubated at 37 °C shaking with 50 rpm for three months. Scaffolds of each degradation period were taken out at different periods of time, washed with distilled water, and vacuum dried for characterization analysis.

### 2.8. Synthesis of UV-Curable GMHA

First, 1 g of hyaluronic acid (Mw = 4.4 × 10^5^) was dissolved in 100 mL PBS and covered overnight under continuous stirring. After it was fully dissolved, 100 mL *N*,*N*-dimethylformamide (DMF), 18.04 mL triethylamine (TEA) and 35.11 mL glycidyl methacrylate (GM) were added separately, in that order, and stirred for 10 days. After that, the solution was then precipitated as a white solid in acetone of 10-fold volume, centrifuged to remove the acetone at 5000 rpm for 10 min. The precipitate was then dissolved in deionized water, and subjected to dialysis (MWCO = 8 kDa) in deionized water for 24 h. Finally, the samples were frozen in −80 °C and freeze-dried to obtain the product.

### 2.9. Isolation and Identification of Mesenchymal Stem Cells

The mesenchymal stem cells (MSCs) were isolated from BALB/c mice. Bone marrow was collected from six-week old BALB/c mice that were sacrificed by carbon dioxide. Their femurs were carefully cleaned of adherent soft tissue and metaphyseal ends of the bones were removed to expose the bone marrow cavity, and bone marrow was harvested through a 0.22 μm filter by flushing with Dulbecco’s modified Eagle’s medium-low glucose (DMEM-LG) supplemented with 10% fetal bovine serum (FBS) and 1% penicillin/streptomycin. MSCs were characterized by positive localization of the multipotent mesenchymal stem cell markers, like CD105, and negative localization of CD34. MSCs should characteristically show positive localization of CD44, CD29, CD105, and CD90, but no localization of hematopoietic markers CD45 or CD34. Flow cytometery could be used to determine the characteristic of MSCs. The cultured MSCs were retrieved by trypsin digestion. Cells aliquots (1 × 10^5^) were washed with fluorescence-activated cell sorting buffer, called stain buffer (2% FBS, 0.1% NaN_3_ in PBS), incubated on ice for 30 min, and stained with fluorescein isothiocyanate (FITC)- and phycoerythrin (PE)-conjugated monoclonal antibodies against mouse CD34 and CD105 (eBioScience). After washing twice with FACS buffer, the cells were fixed with 1% paraformaldehyde (Sigma) in PBS. At least 10,000 events were collected for further analysis using a FACSVantage cytometer and CellQuest software (BD, San Jose, CA, USA).

### 2.10. Cytotoxicity Assay

MSCs were seeded onto scaffolds (length = 10 mm, width = 10 mm and height = 4 mm) and GMHA gel (concentration of 0.25% to 1%) in the 24-well plate at a density of 2 × 10^4^ cells/mL, respectively. Each well was cultured with low-glucose Dulbecco’s Modified Eagle’s Medium (DMEM-LG) with 10% fetal bovine serum (FBS, Gibco) and 1% penicillin/streptomycin from 24 h to 72 h, and cell activity was determined by the MTT (3-(4,5-dimethylthiazol-2-yl)-2,5,-diphenyl-tetrazolium bromide) assay. MTT reagent of 5 mg/mL was added 100 μL to each well, followed by incubation for four hours in an incubator at 37 °C with 5% CO_2_. After removing the medium, 1 mL DMSO was then added to each well to dissolve formazan, and transferred to a 96-well microtiter shaking plate for 15 min, and OD at 570 nm were determined with an ELISA plate reader.

### 2.11. Cell Adhesion and Viability

The MSCs were pre-seeded in each of the scaffolds with 1 × 10^5^ cells/mL in a 24-well culture plate, followed by incubation for four hours in an incubator at 37 °C with 5% CO_2_. After cell seeding, all of scaffolds were induced to undergo growth medium for one month. For staining of cytoskeletons, all of the culture media was removed, adding 4% buffered paraformaldehyde for 30 min, then removed, permeabilized in 0.1% Triton X-100, and incubated with Invitrogen Alexa Fluor^®^ 488 Phalloidin to stain the cytoskeleton protein for 30 min. After incubation, the samples were washed with PBS. Nuclei were stained with 4′6-diamidino-2-phenylindole (DAPI) for 1 min before imaging through the Nikon ECLIPSE Ti-s fluorescence microscope. Cell viability was assessed using an Invitrogen LIVE/DEAD Viability/Cytotoxicity Kit containing approximately 2 μM calcein-AM and 4 μM EthD-1 as a working solution.

### 2.12. Chondrogenic Differentiation

MSCs were pre-seeded in each scaffold with 1 × 10^7^ cells/mL in a 24-well culture plate, followed by incubation for four hours in an incubator at 37 °C, 5% CO_2_. After that, all of the scaffolds were induced to undergo chondrogenic differentiation. Specifically, the constructs were cultured in basic medium as the control consisted of DMEM-high glucose supplemented with 100 nM dexamethasone, 50 μg/mL ascorbic acid, 100 μg/mL sodium pyruvate, 40 μg/mL proline, and 50 mg/mL ITS + 1 liquid media supplement. The chondrogenic differentiation medium was the basic medium with 10 ng/mL recombinant human transforming growth factor-beta 1 (TGF-beta 1; PeproTech Inc., Rocky Hill, NJ, USA). At days 7, 14, 21, and 28, the samples were harvested for subsequent characterization.

### 2.13. Real-Time Polymerase Chain Reaction (qPCR) Analysis of Gene Expression

Total RNA was extracted using the mirVana™ miRNA Isolation Kit (Invitrogen, Carlsbad, CA, USA). Isolated RNA was reverse-transcribed with a High-Capacity cDNA Reverse Transcription Kit (Invitrogen, Carlsbad, CA, USA), and real-time PCR analysis was performed using the ABI 7300 Real-Time PCR System (Applied Biosystems, Foster City, CA, USA) with Power SYBR^®^ Green PCR Master Mix (Invitrogen, Paisley, UK). Real-time PCR conditions were as follows: 95 °C for 8.5 min, followed by 40 cycles of 95 °C for 30 s, 58 °C for 30 s, and 72 °C for 30 s. The relative expression ratio between the target genes and the control group were calculated. The PCR primers were as follows: Sox9: forward, 5′-CGGCTCCAGCAAGAACAAG-3′ and reverse, 3′-TTGTGCAGATGCGGGTACTG-5′; Aggrecan: forward, 5′-AGATGGCACCCTCCGATAC-3′ and reverse, 3′-ACACACCTCGGAAGCAGAAG-5′; Col2a1: forward, 5′-GGAGGGAACGGTCCACGAT-3′ and reverse, 3′-AGTCCGCGTATCCACAA-5′; GAPDH: forward, 5′-GCATTGTGGAAGGGCTCA-3′ and reverse, 3′-GGGTAGGAACACGGAAGG-5′. Transcription levels were normalized to GAPDH.

### 2.14. Biochemical Analysis

Sulfated glycosaminoglycan (sGAG) content was spectrophotometrically determined with a Biocolor^TM^ Blyscan assay kit by 9-dimethylmethylene blue chloride (DMMB) methods. Cell number was determined via extraction of total DNA with a genomic Geno Plus^TM^ DNA extraction miniprep system and quantification of DNA content was determined using a Quant-iT PicoGreen dsDNA Assay Kit. Quantitative total sGAG and total DNA were normalized for differences in each sample.

### 2.15. Animal Study

**Figure 3:**
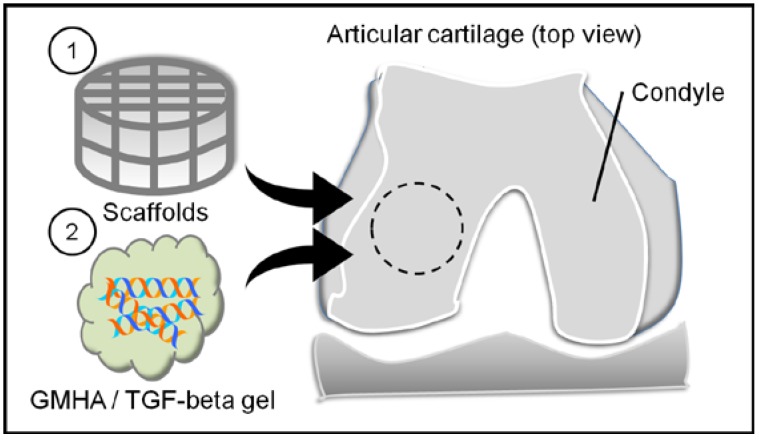
The illustration of animal model. Control group: create a bony defect 3 mm in diameter and 8 mm in depth without scaffold implantation. Experimental group: scaffolds and light-curing gel containing TGF-beta 1 were implanted into the defect, and cross-linked with 365 nm ultraviolet light.

Six male New Zealand white rabbits (eight weeks old) were chosen as the animal model. Rabbits were generally anaesthetized, and had their knees shaved and disinfected. Both knees of each animal were operated during the same surgery, with arthrotomy made through a longitudinal medial parapatellar incision and lateral dislocation of the patella. A 3 mm diameter circle was drilled in the center of the medial femoral condyle and holes were left to bleed. Then, scaffolds and light-curing gel containing TGF-beta 1 were implanted into the defect, cross-linking with 365 nm ultraviolet light for 5 min (Figure 3). Joints were harvested after three months from implantation and evaluated histologically as described below. Joints were fixed in 4% buffered paraformaldehyde for one to two weeks (replaced with fresh 4% buffered paraformaldehyde per three days). After that, joints were demineralized by 10% ethylenediaminetetraacetic acid (EDTA) solution for one month (replaced with fresh 10% EDTA solution per week). When demineralization finished, all of the specimens were dehydrated in increasing grades of ethanol, defatted and cleared with xylene, and embedded in wax.

### 2.16. Statistical Analysis

The results of all experimental data were expressed as means ± standard deviation and compared using Student’s *t*-test. The difference was considered significant when *p* < 0.05.

## 3. Results

### 3.1. Characterization of Diblock Biopolymers

**Figure 4:**
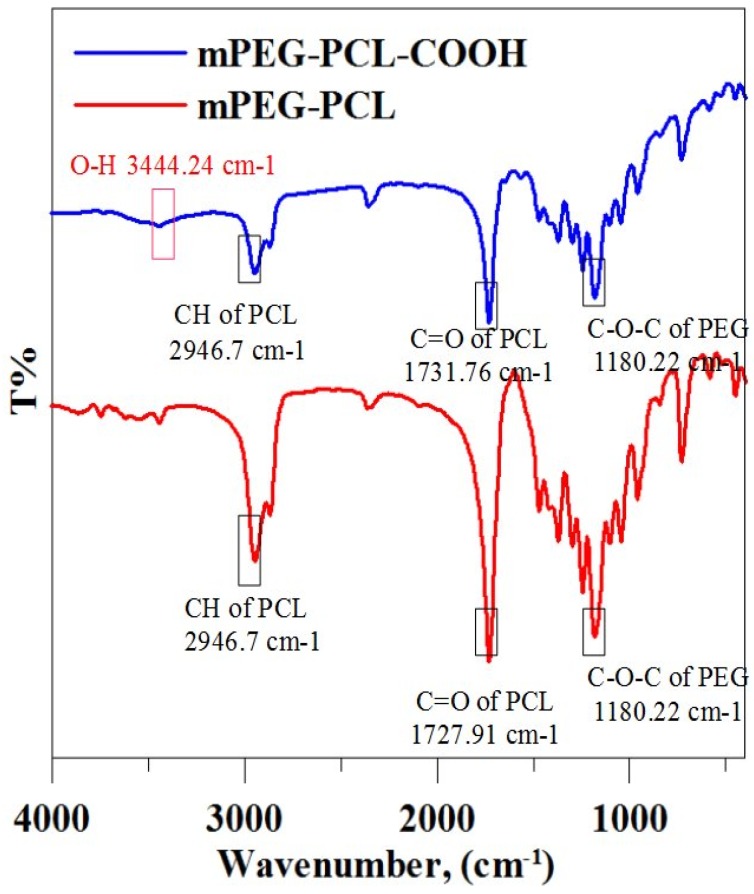
The FTIR spectra of mPEG-PCL and mPEG-PCL-COOH, respectively.

**Figure 5:**
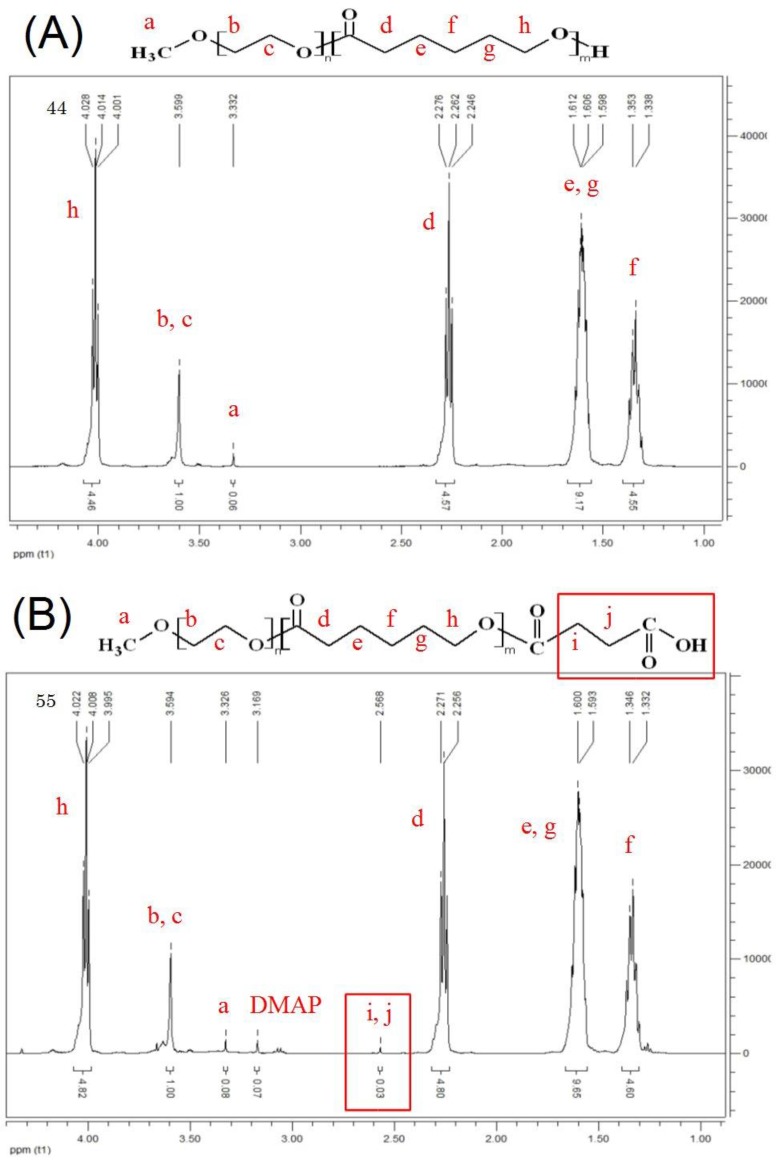
^1^H NMR spectrum of (**A**) mPEG-PCL and (**B**) mPEG-PCL-COOH.

Characteristics were observed between the DSC diagrams of mPEG-PCL and mPEG-PCL-COOH polymers. The melting temperatures of mPEG-PCL and mPEG-PCL-COOH were identified at 60.63 °C and 59.06 °C, respectively. The freezing temperatures were identified at 32.92 °C and 36.34 °C, respectively. These results are in good agreement with the previous study in DSC [34], and no significant differences were observed between the mPEG-PCL and mPEG-PCL-COOH. Therefore, the temperature of the rapid prototyping machine heat module should be higher than 60 °C to prepare the scaffolds. In the FTIR spectra of the biopolymers, a strong absorption band associated with the carbonyl group at 1731.76–1727.91 cm^−1^ was observed. Another peak at 1180.22 cm^−1^ indicated the C–O–C stretching. The FTIR spectrum of mPEG-PCL-COOH showed the presence of hydroxyl (OH–) stretching modes in the carboxyl group at 3444.24 cm^−1^ (Figure 4). Molecular weight and distribution of biopolymers were also characterized by GPC and ^1^H NMR, respectively, and summarized in Table 1. The results of GPC are shown in Table 1. As can be seen, the total molecular weight and polydispersity (PDI, *M*_w_/*M*_n_) of mPEG-PCL-COOH biopolymer is 9514.33 ± 389.70 and 1.19 ± 0.01, respectively. The typical signals of the ^1^H NMR spectra of both mPEG-PCL and mPEG-PCL-COOH were detected as shown in Figure 5, and CDCl_3_ was used as a solvent, thus confirming that the ring opening polymerization and coupling reaction were carried out. As seen in the spectrum of mPEG-PCL, the characteristic peaks at 1.37, 1.62, 2.29, and 4.06 ppm are attributed to the methylene protons of –(CH_2_)_3_–, –OCCH_2_– and –CH_2_OOC– in the PCL unit. The PEG unit shows its characteristic peaks at 3.38 and 3.64 ppm, which are the signals of CH_3_– and –CH_2_CHO–. The spectra of mPEG-PCL-COOH show succinic anhydride ring opening modifications of the carboxyl group at 2.66 ppm. The average molecular weight (*M*_n_) was calculated by using ^1^H NMR spectroscopy, in accordance with the previous study [35].

### 3.2. The Inorganic Component in the Scaffolds

Specimens taken from random spots and weighing approximately 10 mg, were heated from 25 to 900 °C at 10 °C per min. From thermogravemetric analysis (TGA), it was observed that the onset of thermal degradation started at about 300 °C, and at 400 °C the entire amount of PCL had degraded. However, there was no thermal degradation observed in pure HAp, as heating was performed only up to 900 °C, Thermal stability of the HAp was evident from the TGA curve obtained up to 900 °C. In fact, the thermal degradation temperature of HAp is in the range between 1360 °C to 1400 °C [36]. The total weight loss of the HAp sample of about 2 wt % can be attributed to the loss of physically and chemically absorbed water and CO_2_ elimination as a result of the decarbonation process in the range 400 °C to 900 °C. As seen in the TGA of the scaffolds, the weight percentage that was left in the pan indicated the composition of HAp, which was 33 wt %. This is a good indication that the physically blended biocomposite was homogeneously mixed.

### 3.3. Mechanical Properties and Degradation of Scaffolds

**Figure 6:**
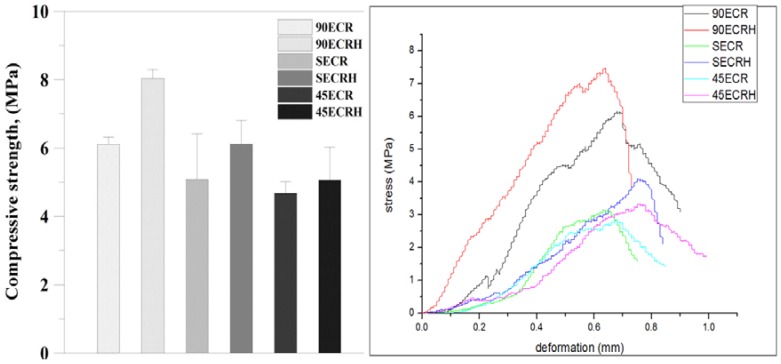
Compressive strength of each structure of scaffolds (three groups of samples were tested).

To investigate the influence of HAp incorporated into the scaffolds on the mechanical properties, compression testing was performed on three different structures (90ECR, 45ECR, and SECR) and HAp impregnation. The compressive strength of three different structures of scaffolds, adding HAp or not, is shown in Figure 6. The compressive strength of 90ECRH (with a lay-down patterns of 0°/90°) structure is the highest, which was similar to articular cartilage [37]. Therefore, 90ECRH was chosen for the following experiments. In the degradation test, scaffolds were immersed in PBS at 37 °C, shaking at 50 rpm to imitate the human articular environment for three months. It is well-known that proper degradation in a physiological environment is one of the most important characteristics of a scaffold in tissue engineering. The degradation behavior of the scaffolds was evaluated from the weight loss and morphological changes. The incorporation of time increases the degradation of the scaffolds. After 12 weeks of degradation, sample weight lost 85 wt %, compressive and Young’s modulus decreased from 7.15 MPa to 2.47 MPa and from 120.92 MPa to 75.76 MPa, respectively. the half-life of the scaffold is over 60 days.

### 3.4. Scanning Electron Microscope Imaging

The SEM image of materials and scaffold before degradation and after degradation were scanned by Hitachi TM-1000. In the image of scaffolds before degradation, there is a smooth surface of fiber. After 12 weeks of immersion in PBS, scaffolds exhibited the rough surface with HAp exposure. The image of the scaffolds clearly illustrates the white dots on the fracture and erosion surface, and homogeneously distributed inside the scaffolds. According to the fracture and erosion surfaces, the scaffold underwent surface erosion.

### 3.5. Extraction and Identification of MSCs

In this study, all of the mesenchymal stem cells were isolated from four-weeks old BALB/c mice from BioLASCO. The MSCs positively expressed CD29, CD44, CD105, and Sca-1. Nevertheless, they were negative for CD3, CD19, CD11b, CD45, CD117, CD34, TER-119, CD86, H-2kb, and I-Ab [38]. We chose FITC-conjugated CD34 and PE-conjugated CD105 to confirm cell surface markers by BD FACSCalibur. Over 80% of the cells showed red fluorescence (CD105-PE) and only 2% of cells showed green fluorescence (CD34-FITC). Therefore, the cells were identified to be MSCs isolated from the femurs of BALB/c mice.

### 3.6. Cytotoxicity

**Figure 7:**
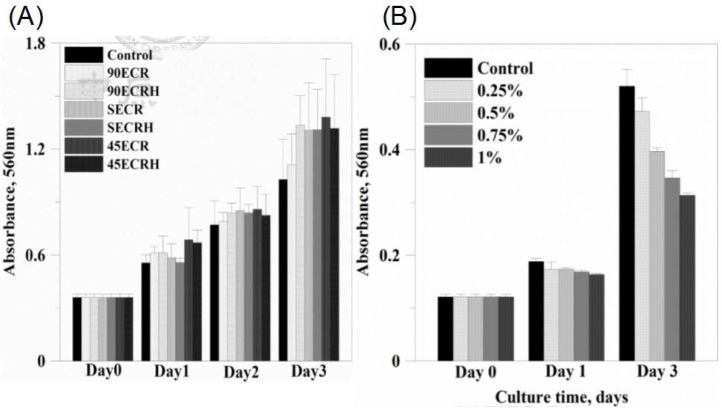
Cytotoxity of (**A**) scaffolds and (**B**) GMHA by MTT assay. Error bars represent means with standard deviation.

Cytotoxicity of the scaffolds and synthesized gels were assessed by MTT assay. The cells have been seeded on the scaffold or on the gel to co-culture for three days. Figure 7A shows the proliferation behavior of MSCs on the surface of scaffolds after cells were seeded and cultured from day 1 to day 3 under standard conditions. Cell viability increased as culturing time increased. After three days culturing, the O.D. value increased one-fold in comparison to day 1. In Figure 7B the cell viability of GMHA gel is shown. There is a slight cytotoxicity in higher concentrations of GMHA; nevertheless, the O.D. value increased as the culturing time increased. Therefore, for the in vivo study, we chose 1% GMHA gel to encapsulate the growth factor.

### 3.7. Cell Adhesion and Cell Viability

**Figure 8:**
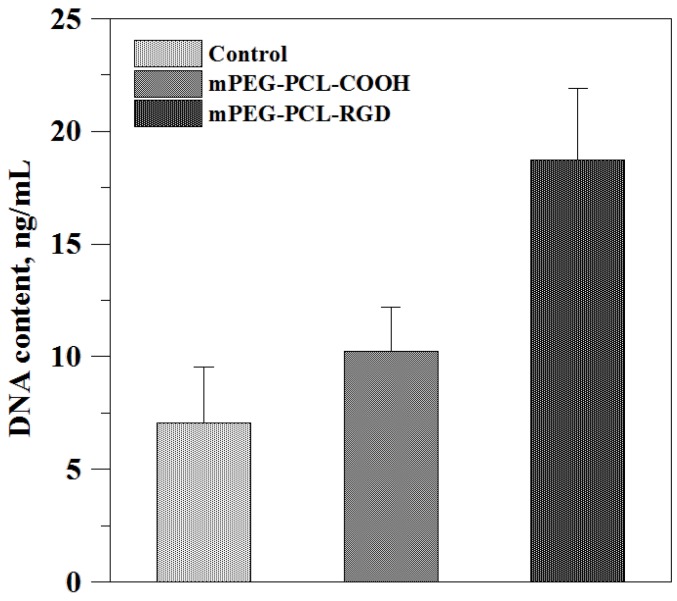
The DNA content of cell adhesion on 2D materials. Three days after cell culture, DNA detection showed that grafting RGD peptide in the material can effectively enhance cell attachment and proliferation.

**Figure 9:**
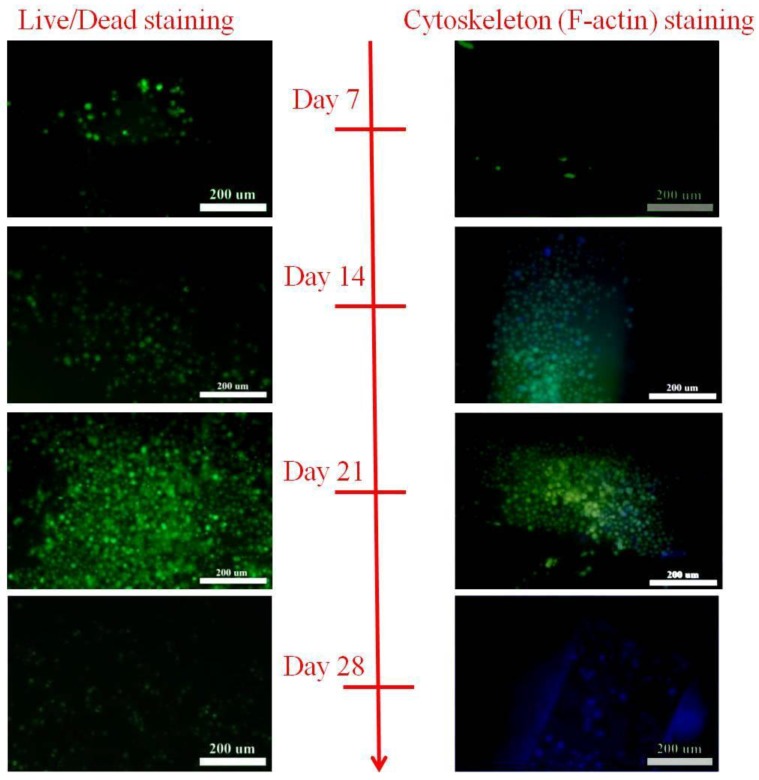
The phalloidin staining of the F-actin and LIVE/DEAD staining of cells on 3D scaffolds after 7, 14, 21, and 28 days of culturing.

The morphology and cytoskeleton organization of the cells on the constructs was analyzed after phalloidin staining of the F-actin. For cells adhesion on 2D slides coated with materials, RGD peptide grafting could enhance the formation of F-actin which appeared as a green fiber inside the cells. The DNA content of cells also showed higher quantification in comparison to un-grafted and control groups (Figure 8). In 3D culturing, obviously, cells increased significantly with the increase of the culture time on the constructs, and cell viability was good according to the LIVE/DEAD staining, as shown in Figure 9. The DNA content of cells showed the highest quantification on day 21.

### 3.8. Gene Expression

**Figure 10:**
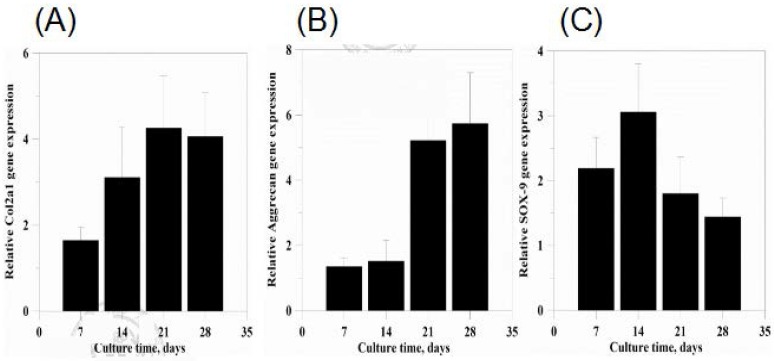
Chondrogenic differentiation of MSCs in aggregate culture following transduction. Relative expression of mRNA of (**A**) type II collagen; (**B**) aggrecan; and (**C**) Sox9 of MSCs cultured in vitro for up to 28 days. mRNA expression levels were normalized to the housekeeping gene GAPDH and control group.

To evaluate the chondrogenic and hypertrophic differentiation of MSCs, real-time polymerase chain reaction (qPCR) was performed for selecting chondrogenic markers (type II collagen, aggrecan, and Sox9). Gene expressions were normalized to GAPDH and to cells culturing with growth medium of selected chondrogenic markers. Figure 10 shows the relative gene expression of Col2a1 (Figure 10A), aggrecan (Figure 10B), and Sox9 (Figure 10C), respectively. MSCs seeded on scaffolds, culturing with chondrogenic differentiation medium showed higher aggrecan gene expression on day 28, higher Col2a1 gene expression on day 21, and higher Sox9 gene expression on day 14, respectively.

### 3.9. sGAG Content

**Figure 11:**
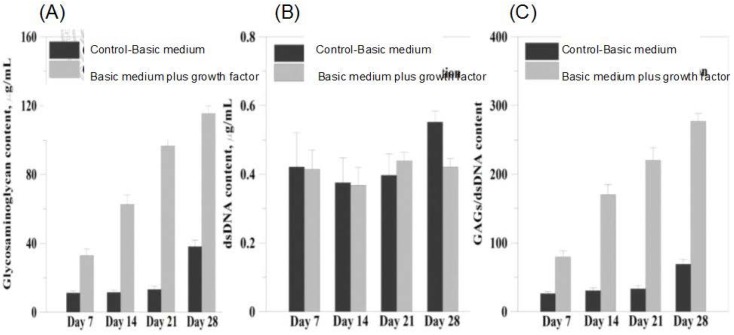
Biochemical analysis of MSCs differentiation. (**A**) sGAG contents of the MSCs after 7, 14, 21, and 28 days of differentiation; (**B**) DNA contents of the MSCs after 7, 14, 21, and 28 days of differentiation and (**C**) normalized s-GAG contents with respect to DNA contents.

MSCs could differentiate into chondrocytes in the presence of transforming growth factor -beta and dexamethasone. In our study, chondrogenic differentiation of MSCs was induced by chondrogenic differentiation medium containing transforming growth factor-beta, dexamethasone, and 1% ITS + 1 supplement for four weeks. Sulfated glycosaminoglycan (sGAG) quantification analysis was performed by using the Blyscan™ Glycosaminoglycan Assay kit. After four weeks of culture, Figure 11 showed the content of sGAG normalized to the content of DNA, culturing in the chondrogenic differentiation medium has a higher level compared with that cultured in the growth medium. The sGAG/DNA ratio increased significantly as the culture time increased.

### 3.10. Histological Analysis

**Figure 12:**
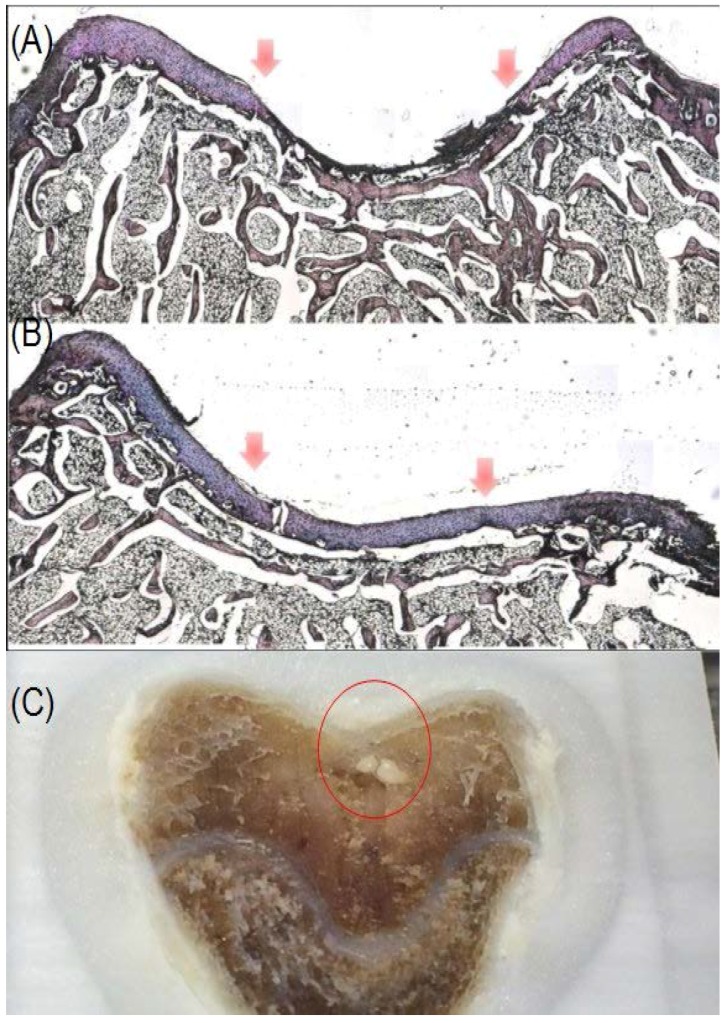
The histological staining (Alcian blue/PAS staining) and specimen of cartilage sections of rabbit after 12 weeks after implantation. (**A**) The control group with only bone formation; and (**B**) the experimental group showed bone and hyalin cartilage regeneration (arrow means defect); (**C**) The specimen of the experimental group showed good cartilage and bone regeneration on the surface layer and residual un-degraded scaffold in the deep layer.

In histological analysis, rabbit articular cartilage sections were stained with Alcian blue and periodic acid-schiff (PAS) double-staining with a Polyscience Alcian Blue/PAS kit. Alcian blue staining indicated sGAG accumulation, imparting a blue color to the acidic mucins and other carboxylated or weakly sulphated acid mucosubstances. Periodic Acid Schiff reaction was then used to stain the basement membranes, glycogen, and neutral mucosubstances pink to red. Mixtures of neutral and acidic mucosubstances will appear purple due to positive reactions with both Alcian Blue and PAS. Figure 12 shows sections stained by Alcian Blue and PAS double staining, after 12 weeks scaffolds and GMHA gel containing TGF-beta 1 were implanted. Articular cartilage and subchondral bone were healed in comparison to the control group, which showed only bone formation.

## 4. Discussion

Tissue engineering and modelling of native articular cartilage in vivo requires an appropriate density and function of chondrocytes in a three-dimensional environment. Many studies have reported how one or more materials affect osteoarthritis, however, in those cases, their research should isolate primary cells from volunteers or patient-self to pre-culture with materials before implanting [3,39,40]. In our study, we proposed MSCs mixing with the implant by capillarity so that we do not need to pre-culture the chondrocytes with the implant before using. Therefore, scaffolds play an important role in directing 3D tissue regeneration [41]. According to Woodfield et al. [13] and Wang et al. [7], traditional processing could not control the intra-structure of the scaffold. Using RP, it is possible to control the critical factors affecting tissue regeneration within the scaffold, like pore size and interconnection. Pore size of 300 μm is better for MSCs proliferation and chondrogenic differentiation [42], and a high interconnection of the scaffold could result in capillarity and homogeneous distribution of cells. Adding HAp to the prototyped scaffold could increase the mechanical properties. The array type of the filament could influence the structure properties, as well. In our experiments, compositions of polymers and HAp with a weight ratio 2:1, and laying down patterns 0°/90° significantly increased the compressive strength up to 30% (Figure 6). The compressive strength of the scaffold is 7.15 Mpa, which is consistent with the values reported by De Santis et al. [43] for rapid-prototyped hydroxyapatite-reinforced PCL/PEG scaffolds, ranging between 5.7 MPa and 13 MPa. The Young’s modulus of our RP HAp-reinforced scaffold is 120.92 Mpa, also similar to the value reported by De Santis et al., ranging between 41 MPa and 125 MPa. The values are higher than those without HAp reinforcement, as reported by Wang et al. [31].

In in vitro studies, MSCs could adhere, proliferate, and differentiate on the scaffold biomanufactured through RP. TGF-beta will activate TGF-β receptor II on the cell surface and then bind with TGF-β receptor I, inducing TGF-β receptor II and TGF-β receptor I to combine in a heteromeric complex, activating the downstream Smad pathway [44,45]. In gene expression of chondrogenic differentiation, Sox (Sry-type high mobility group box) acts as a DNA-binding protein involved in the regulation of sex determination, bone development, and neurogenesis formation in the animal development process [46]. Sox9 is one of the transcription factors in the Sox family involved in the regulation of bone development. It has a transcriptional activation domain in the high-mobility-group-box DNA-binding domain, combining with a special sequence of minor DNA grooves. In the process of chondrogenic differentiation, Sox9 will bind chondrocyte-specific enhancers of Col2a1, activating the target gene of chondrogenic differentiation [47,48]. MSCs cultured with appropriate conditions, affecting growth factor TGF-beta, the Sox9 gene will express in the early stage of chondrogenic differentiation (Figure 10C) [49]. The Sox9 gene can also enhance aggrecan promoter activity [50], that is, aggrecan gene expression is slower than Sox9 gene expression (Figure 10B).

In in vivo studies, we reported the successful use of RP scaffolds infiltrated with GMAH gel containing TGF-beta for regeneration of articular cartilage defects in rabbits. We implanted RP scaffolds infiltrated with GMHA gel containing TGF-beta into osteochondral defects in rabbits, without exogenous cell seeding. These scaffolds could recruit endogenous bone marrow MSCs for chondrogenesis and facilitate hyaline cartilage regeneration. There are three types of cartilage (hyaline cartilage, fibrocartilage, and elastic cartilage) [51]. Compared to other types of cartilage, hyaline cartilage has fewer fibers in its matrix, and microscopy investigations show that this type of cartilage looks very smooth. After 12 weeks from implantation, sections were stained by Alcian blue/PAS, and we found that cartilage and subchondral bone were healed in the implanted group; nonetheless, we found no cartilage formation in the control group (Figure 12). RP scaffolds can promote bone healing as is known from our previous study [10]; nevertheless, this is our first time to promote cartilage regeneration. The new formation of cartilage is hyaline cartilage, and the fibrocartilage was composed of a fibrous layer, proliferative layer, hypertrophic layer, and mineralized layer. The hyaline cartilage had a homogeneous structure, and consisted simply of a cartilaginous matrix and chondrocytes [52].

Unlike other studies in cartilage regeneration [13,17,25,31], our study integrated required factors for cartilage growth. We used FDM to fabricate a biodegradable porous PCL scaffold, modified by RGD peptide grafting for cell adhesion, and light-curing HA gel containing TGF-beta 1 on the surface for cartilage regeneration. This biphasic scaffold can offer both mechanical support and a biological environment for bone and cartilage regeneration.

## 5. Conclusions

In our study, we showed that a rapid-prototyped scaffold infiltrated with UV light-cured hyaluronic acid hydrogel containing growth factor TGF-β1 could enhance the healing of the osteochondral defect in the knees of rabbits. This approach showed good integration of scaffolds coupled to hyaline cartilage repair tissue without damaging the adjacent native articular cartilage. The osteochondral defect was filled with the bio-composite and biodegradable implant, blood with mesenchymal stem cells was added to the implant by capillarity, and then differentiated into chondrocytes on it. Compared to current treatments of osteoarthritis, our study proposed a new clinical option for a surgical treatment of osteoarthritis.

## Figures and Tables

**Table 1:** *M*_n_, *M*_w_, and PDI (*M*_n_/*M*_w_) of mPEG-PCL determined by GPC and ^1^H NMR.

Number average molecular weight (*M*_n_) of NMR	12546.07 Da
Number average molecular weight (*M*_n_) of GPC	7969.67 ± 289.0144 Da
Weight average molecular weight (*M*_w_) of GPC	9514.33 ± 389.6977 Da
Polydispersity (PDI) of GPC	1.19 ± 0.0061
